# Length-Dependent Deep Learning Model for RNA Secondary Structure Prediction

**DOI:** 10.3390/molecules27031030

**Published:** 2022-02-02

**Authors:** Kangkun Mao, Jun Wang, Yi Xiao

**Affiliations:** School of Physics and Key Laboratory of Molecular Biophysics of the Ministry of Education, Huazhong University of Science and Technology, Wuhan 430074, China; mkk@hust.edu.cn (K.M.); junwang@hust.edu.cn (J.W.)

**Keywords:** RNA secondary structure, deep learning, length-dependent model

## Abstract

Deep learning methods for RNA secondary structure prediction have shown higher performance than traditional methods, but there is still much room to improve. It is known that the lengths of RNAs are very different, as are their secondary structures. However, the current deep learning methods all use length-independent models, so it is difficult for these models to learn very different secondary structures. Here, we propose a length-dependent model that is obtained by further training the length-independent model for different length ranges of RNAs through transfer learning. 2dRNA, a coupled deep learning neural network for RNA secondary structure prediction, is used to do this. Benchmarking shows that the length-dependent model performs better than the usual length-independent model.

## 1. Introduction

Non-coding RNAs play a lot of roles in biological activities, from transcriptional regulation to guiding modification [1,2]. To understand their biological functions, it is preferable to know their tertiary structures. Currently, experimental determination of RNA tertiary structures is still difficult, and only about 2000 RNA monomer structures have been measured. Therefore, many theoretical or computational methods have been proposed to predict RNA tertiary structures [3,4,5,6,7,8,9,10,11]. Most of these methods use secondary structures as their input. Therefore, the ability to predict tertiary structures depends on whether the secondary structures can be correctly predicted.

Popular traditional methods for predicting RNA secondary structures can be classified into single-sequence methods and homologous-sequence methods. Single-sequence methods are those folding algorithms with thermodynamic, probabilistic or statistical scoring schemes by applying the minimum free energy principle, such as Mfold [12], UNAfold [13], RNAfold [14], and RNAstructure [15]. Compared to the single-sequence method, homologous-sequence methods determine base pairs conserved among homologous sequences, such as TurboFold [16]. Homologous-sequence methods require a large number of homologous sequences.

Recently, deep learning has made impressive progress across a variety of fields, including bioinformatics, such as RNA secondary structure prediction; for example, DMFold [17] predicts the dot or bracket state by bidirectional long short-term memory (LSTM) at first and then infers the base pair using an algorithm based on the improved base pair maximization principle. SPOT-RNA [18] treats the entire RNA secondary structure as a two-dimensional contact matrix, and uses an ensemble of deep hybrid networks of ResNets coupled with 2D-bidirectional LSTMs to predict. E2Efold [19] is a two stage end-to-end deep learning model; the first part uses an attention mechanism to output a symmetrical base pairing score matrix, and the second part enforces the constraints and restricts the output space. Additionally, MXfold2 [20] introduces a deep neural network into an RNA folding score function to learn Turner’s nearest-neighbour free energy parameters. We proposed a deep learning method to improve RNA secondary structure prediction using direct coupling analysis of aligned homologous sequences [21]. Recently, we also proposed an RNA secondary structure prediction method with pseudoknots named as 2dRNA [22], which used coupled deep learning neural networks of bidirectional LSTM [23] and U-net [24], and was trained, validated and tested by using the dataset ArchiveII [25]. These deep learning methods showed better performance than traditional prediction methods. However, their prediction accuracies still leave much room for improvement.

RNAs have very different lengths, usually from tens to thousands of nucleotides, and their secondary structures are very different too. Therefore, it is difficult for the deep learning model to learn the secondary structures of RNAs with very different lengths. It is expected that those with similar lengths may be more easily learned. In the present work, we report an improved version of 2dRNA, with a length-dependent model (2dRNA-LD) that is trained for different length ranges of RNAs using transfer learning. Furthermore, we use a larger dataset of bpRNA and apply a grid search for the hyperparameters of the network. The results show that the length-dependent model (2dRNA-LD) can further improve the prediction performance of the length-independent model (2dRNA-LID).

## 2. Results and Discussion

We have benchmarked 2dRNA-LD on a bpRNA-based testing set, TS0 [18]. Since SPOT-RNA [18] has been compared with 12 other available RNA secondary structure prediction methods, such as mxfold [26], ContextFold [27], CONTRfold [28], IPknot [29], RNAfold [14] and RNAstructure [15], here we only compare 2dRNA with SPOT-RNA. In addition, here, we also compare two other recent methods of using deep learning, E2Efold [19] and MXfold2 [20]. However, we note that the training dataset of E2Efold is RNAStralign [16], but it is different from the bpRNA-based dataset, so these results are only for reference and they cannot be compared. MXfold2, on the other hand, provides multiple pre-trained models that include a bpRNA-based training set, TR0.

### 2.1. Length-Independent Model

We first show the performance of 2dRNA with a length-independent model (2dRNA-LID). This model is different from the previous 2dRNA [22], in that it was trained in the larger training set, TR0, of bpRNA, instead of ArchiveII, and is an ensemble of the top five models obtained by grid searching for the hyperparameters of the network. Table 1 shows the prediction performance of 2dRNA-LID on the TS0 testing set. We also list the corresponding result of SPOT-RNA, which is from the SPOT-RNA paper [18]. The MCCs of 2dRNA-LID are 0.611, 0.648 and 0.659 when the pairing cutoffs are 0.5, 0.3 and 0.1, respectively. When the cutoff is 0.3, 2dRNA-LID has a better balance of PPV and STV.

### 2.2. Length-Dependent Model

**Figure 1:**
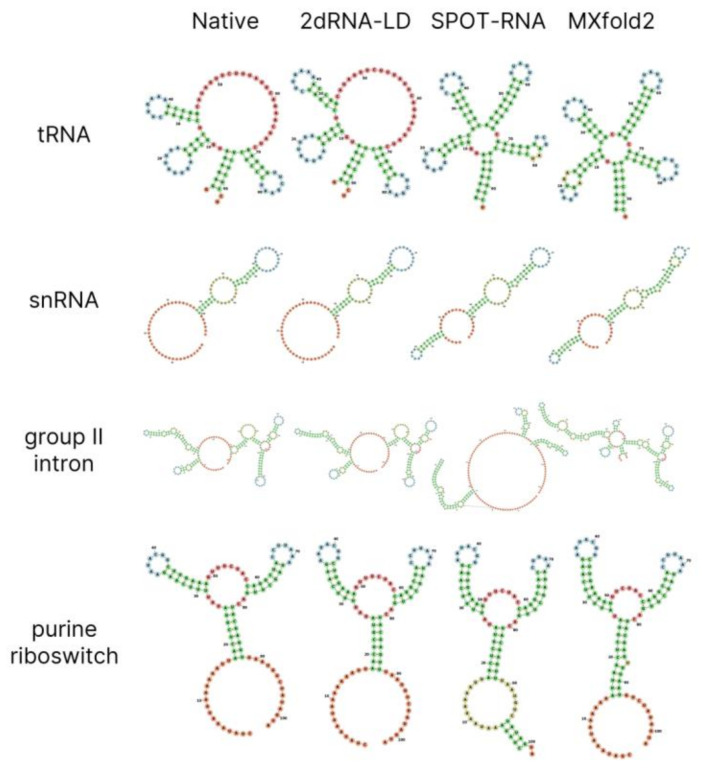
Comparison of 2dRNA-LD and other methods of prediction with the native structure of a tRNA, snRNA, group II intron, and purine riboswitch from testing set TS0. The secondary structure of a tRNA (bpRNA_RFAM_1243), snRNA (bpRNA_RFAM_24262), group II intron (bpRNA_RFAM_38453), and purine riboswitch (bpRNA_RFAM_8591) is represented by a 2D diagram, which is plotted by the Forna webserver. The nucleotides are coloured according to the type of structure that they are in, as follows: stems (green), multiloops (red), interior loops (yellow), hairpin loops (blue), and 5′ and 3′ unpaired regions (orange).

Since the lengths of the RNAs in the training set, TR0, used in initial training were very different, ranging from 33 to 498 nucleotides, it was easier for the neural network model to learn the secondary structures of sequences with similar lengths. Therefore, we divided the RNAs in the TR0 training set according to their sequence lengths into five length intervals in steps of 100 nucleotides, and then performed transfer learning on each of them using the top five length-independent models trained on the whole TR0 set. From Table 2, we can observe that the MCCs were improved in every length interval in comparison with those of the length-independent model. In particular, those for long-length intervals improved considerably, from 0.527 to 0.591 for 301∼400, and from 0.441 to 0.508 for 401∼500. As a result, the average STY, PPV and MCC of the length-dependent model are improved for all cutoff values (Table 1 and Table S8). As shown in Table 1, the MCCs of 2dRNA-LD increased to 0.672 when the pair cutoff was 0.3. Compared with SPOT-RNA, both 2dRNA-LID and 2dRNA-LD showed better performance in STY, PPV and MCC for the pairing cutoff of 0.3. It is noted that the default value of the pair cutoff in the released SPOT-RNA is 0.335. In addition, the prediction of 2dRNA-LD (cutoff = 0.3) and other methods was compared with the native structure across different types of RNA, as shown in Figure 1.

We also selected three other methods (IPknot, RNAfold and SPOT-RNA) for comparison. Table 3 shows the results for the different length intervals. It can be observed that the performance of other methods gradually decreased as the length increased, but our length-dependent model 2dRNA-LD avoids this; as the length increased, it did not lose much performance, especially on PPV and MCC.

### 2.3. Prediction of Pseudoknot Base Pairs

The output of our method is an L×L×1 matrix, which also includes information on pseudoknot base pairs. In the TS0 testing set, there are 129 structures, with a total of 1206 pseudoknot base pairs, and the result is shown in Table 4. 2dRNA-LD (cutoff = 0.3) shows better performance in pseudoknot base pairs than 2dRNA-LID and SPOT-RNA, with 569 pseudoknot base pairs being correctly predicted out of a total of 1206. In contrast, 2dRNA-LID and SPOT-RNA only predicted 334 and 282 pseudoknot base pairs, respectively.

### 2.4. Non-Canonical Pairs

The bpRNA dataset also included information about non-canonical base pairs. Since the output L×L×1 matrix of our model includes the probabilities of base pair formation of any two residues, the prediction can also include non-canonical base pairs, as well as the canonical Watson-Crick (A-U and G-C) and Wobble (G-U) base pairs between residues. The prediction results for TS0 are shown in Table 5. It is shown that the performances can be even better than those without non-canonical base pairs.

### 2.5. Discussions

**Figure 2:**
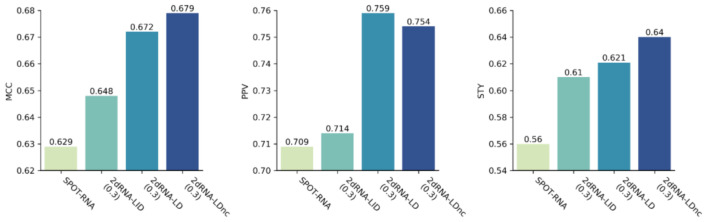
The performance of SPOT-RNA, 2dRNA-LID, 2dRNA-LD and 2dRNA-LDnc on the testing set TS0.

From the above results, we can observe that the length-dependent model performed better than the length-independent model (also see Figure 2). To understand this result, we discuss some factors that may have affected the models’ performances.

#### 2.5.1. Sequence Number and Length Distribution

**Figure 3:**
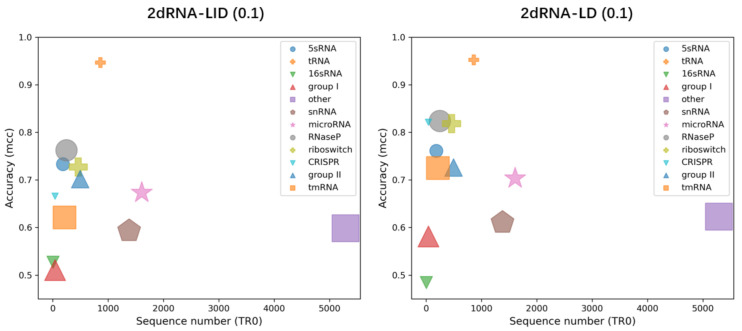
Dependence of the performance of 2dRNA-LID and 2dRNA-LD for each type of RNA in the training set TR0 on its sequence number and the width (longest to shortest) of its length distribution. The width of the length distribution of each type of RNA is proportional to the size of the symbol denoting it.

We first analysed the performance dependence of 2dRNA-LID and 2dRNA-LD on the sequence number and length distribution of different types of RNAs in the training set TR0. To do this, we made predictions using 2dRNA-LID and 2dRNA-LD for different types of RNAs in the TS0 set (see Table S7, Supplementary Materials). This demonstrated that the performance for each type of RNA depends on not only the sequence number, but also the length distribution of this type of RNA in the training set, which can be intuitively observed from Figure 3. When the sequence lengths have a wide distribution, the performance is very low, even though the sequence number is very large. This may explain why the length-dependent model performed better than the length-independent model. Figure 3 shows that the performance of 2dRNA-LID is significantly improved by 2dRNA-LD.

#### 2.5.2. Top Five Models

We applied a grid search to optimize the hyperparameters of 2dRNA, such as the number of LSTM layers, dropout rate, learning rate, and so on. The top five performing models out of 320 trained models were selected by the validation set VL0. Table 6 shows their performances on the VL0 and TS0 sets, and their hyperparameters are given in Table 7. The final model is assembled from the top five models, and the output is given by averaging the results predicted by these five models. From the results, we can observe that no single model had an MCC value exceeding 0.6, but combining the top five models could achieve up to 0.61 for MCC, which mainly relies on the improvement of PPV from 0.677 to 0.725.

#### 2.5.3. Pairing Cutoff

The final output of our method is an L×L×1 matrix, which is normalized into $[0,1]$ through the sigmoid layer, indicating the pairing probability of each pair of bases. Two residues will be considered to form a base pair if the value of the corresponding pairing probability is greater than the pairing cutoff. Table 1 gives the results for three different cutoffs. It can be observed that the PPV decreases, while the STY and MCC increase with the decrease in the cutoff. Therefore, if we need high PPV, we can use a larger cutoff (0.5), and if we need high MCC or STY, we can use a small cutoff (0.1). The cutoff 0.3 gives a good balance between PPV and STY, i.e., improving STY significantly, without losing too much PPV.

#### 2.5.4. Transfer Learning for TS1_Neat

The final model of SPOT-RNA is a deep learning model after transfer learning on a PDB dataset, TR1 [18]. Since the number of RNAs in the PDB dataset is very limited, we only give a brief discussion. We performed similar transfer learning on the dataset TR1_neat, which is the TR1 processed by RNApdbee (see datasets in the Methods section). With the initial training on TR0, and then transfer learning on TR1_neat, the testing of our length-independent model (2dRNA-LID-PDB) on TS1_neat shows higher PPV, but lower STY, than SPOT-RNA for all the three cutoffs, and the MCC is similar to that of SPOT-RNA when the cutoff is 0.1 (Table 8).

We also trained a length-dependent model (2dRNA-LD-PDB) on TR1_neat. However, since the training set TR1_neat only has 120 RNA sequences, and there are 104 sequences whose length is less than 100 (13 sequences for 100∼200, 1 sequence for 300∼400, and 2 sequences for 400∼500), there are not enough data for training the length-dependent model for all the length intervals. Therefore, we trained the length-dependent model for the length interval of less than 100 nucleotides only. The testing results of 2dRNA-LD-PDB on TS1_neat and its subset, with a length less than 100, are shown in Table 8. These results show that the length-dependent model can further improve the performance of the length-independent model, even though the number of RNAs in the PDB dataset is limited. Again, 2dRNA-LD-PDB shows higher PPV and lower STY than SPOT-RNA for the three cutoffs, and the MCC is about 1% larger than that of SPOT-RNA when the cutoff is 0.1. 2dRNA-LD-PDB is also tested on TS0 (see Table S9, Supplementary Materials).

## 3. Materials and Methods

In this paper the secondary structure of an RNA is defined as the pattern formed by the Watson-Crick (A-U and G-C) and Wobble (G-U) base pairs. There are the following two representations for secondary structure: one is dot-bracket notation [30] and the other is dot-plot matrix representation [31]. In dot-plot matrix representation, each base pair $\left(i,j\right)$ is represented by a dot in row i and column j of a rectangular grid or contact matrix of the structure.

### 3.1. Datasets

To evaluate our method, we used the same training, validation and testing datasets, named TR0, VL0 and TS0, as those used in SPOT-RNA [18], and the number of sequences is 10,814, 1300 and 1305, respectively. The TR0, VL0 and TS0 datasets contain different types of RNAs, such as 5sRNA, RNaseP, tRNA, riboswitch and so on, and the sequence length ranges from 33 to 498; more details can be observed in Tables S1–S3 (Supplementary Materials). These datasets are from bpRNA-1m [32], which has 102,348 RNA sequences from seven different sources, including 2588 families from Rfam 12.2 [33] and others including CRW [34], PDB [35], tmRNA [36], SRP [37], etc., then through pre-processing of CD-HIT-EST [38] with a cutoff of 0.8 and the sequence lengths being limited to a maximum of 500 nucleotides. All the sequences have well-annotated secondary structures, including pseudoknot base pairs. Most of the base pairs are canonical base pairs A-U and C-G, and the wobble base pair G-U, but there are also some non-canonical base pairs.

The lengths of RNAs in the training set TR0 were very different. For efficient learning of the deep learning models, we also divided TR0 into five intervals, according to sequence length, in steps of 100 nucleotides. We used this dataset for further transfer learning.

TS1 was another dataset extracted from PDB [35], also provided by SPOT-RNA [18]. As described above, this dataset was preprocessed by CD-HIT-EST [38] with an 80% identity cutoff, followed by BLAST-N [39] with an e-value cutoff of 10 to remove homologous sequences. After this, 217 sequences remained, which were then split into training (TR1), validation (VS1) and testing (TS1) sets, with 120, 30, and 67 sequences, respectively. Because our method needed to use dot-bracket notation of secondary structures in the training process, we used RNApdbee [40] to remove confused base pairs in which one base could participate in more than one base pair and two bases were separated by less than three bases along a sequence to obtain a well-annotated secondary structure. The final training, validation and testing sets are called TR1_neat, VL1_neat and TS1_neat, respectively. See Tables S4–S6 (Supplementary Materials) for detailed information about these datasets.

### 3.2. Pipeline

#### 3.2.1. 2dRNA Model

**Figure 4:**
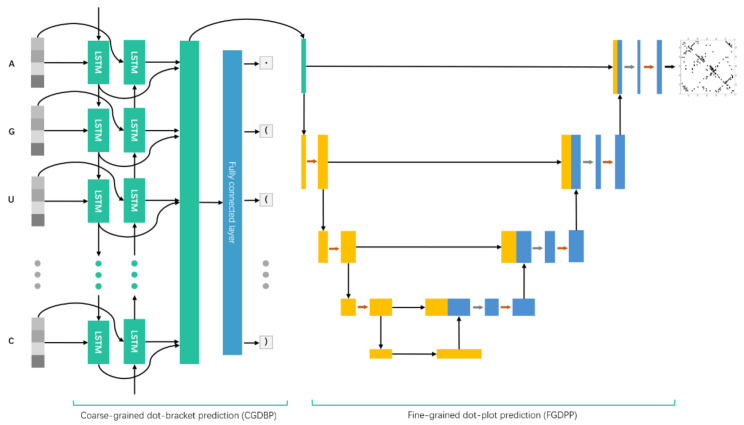
The coupled neural network architecture of 2dRNA. Coarse-grained dot-bracket prediction (CGDBP) uses two-layer bidirectional LSTM and a fully connected layer to output dot-bracket prediction, in which the input is an RNA sequence and the long green box represents hidden vectors from the Bi-LSTM layer. Fine-grained dot-plot prediction (FGDPP) uses U-net to predict pairwise base pairing as the final result, in which the orange and blue boxes are convolutional layers, and the input is hidden vectors from the Bi-LSTM layer (green box).

2dRNA is a coupled two-stage neural network model, including coarse-grained dot-bracket prediction (CGDBP) and fine-grained dot-plot prediction (FGDPP) (Figure 4) [22]. The CGDBP part uses bidirectional LSTM architecture [23] as the encoder, and the FGDPP part uses U-net architecture [24] as a decoder. The input of the network is an RNA sequence of length L, and each residue is obtained by one-hot encoding with L×4 vectors. The LSTM takes this one-hot vector as the input and encoders it into d-dimensional hidden vectors. This L×d embedding performs a pairwise addition operation to obtain a tensor with the size L×L×d, which is then entered into U-net. It can also be input into a fully connected layer to an output dot or bracket state of a base. The final output of 2dRNA is an L×L×1 matrix, which is passed through the sigmoid layer to be normalized into [0,1], indicating the pairing probability of each pair of bases. Further details about these two stages can be found in our previous work [22].

#### 3.2.2. Hyperparameter Search

Although most popular and successful model architectures are designed by human experts, without fine-tuning the hyperparameters that are used to control the learning process, the model still cannot reach its maximum performance. Understanding how to optimize the hyperparameters is still a tough problem for a learning algorithm. There are many approaches to handling this, such as grid search, random search, Bayesian optimization, gradient-based optimization and evolutionary algorithms [41].

Here, we apply a grid search to optimize our model hyperparameters. The hyperparameters include the number of LSTM layers, the size of LSTM hidden vectors, the base channels of the U-net, dropout rate, learning rate and batch size, where the number of the LSTM layer is from 2 to 5, the dimensions of the LSTM hidden vector are selected from 64 and 128, the base channels of the U-net are selected from 16 and 32, the dropout rate is either 0.1 or 0.2, the learning rate varies from 0.002 to 0.02, and the batch size of the training data is 8 or 16. We combined these hyperparameters and performed a grid search over a total of 320 models. After training all the models, we used the validation set VL0 to select the five models with the top performance. Then, the final model was ensembled by the top five models, and this is called the length-independent model (2dRNA-LID). The output was given by averaging the results predicted from these five models.

#### 3.2.3. Transfer Learning

**Figure 5:**
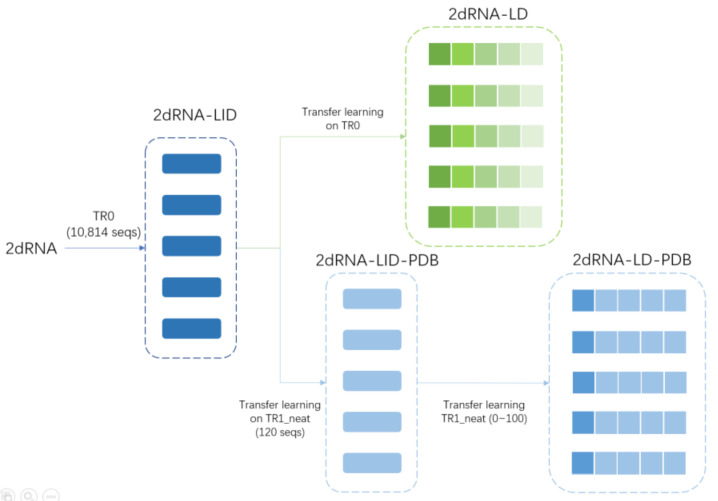
Training procedure of 2dRNA. Each box on the diagram represents a model; the long rectangle in blue/light blue represents the length-independent model, and green/blue squares represent the length-dependent model, in which the different scales of transparency indicate the model trained in different length intervals.

Transfer learning is often used where a model developed for a task is reused as the starting point for a model for a second task. Here, our model was trained in different steps and ways (Figure 5). The model was first trained on the dataset TR0, and the trained model was named 2dRNA-LID. Since the RNAs in the training set TR0 used in initial training had very different lengths, from 38 to 498 nucleotides, and their secondary structures were very different, it is better for the deep learning model to learn the secondary structures of RNAs with similar lengths. Therefore, we divided the training set TR0 according to sequence lengths into five length intervals in steps of 100 nucleotides. At first, we performed transfer learning on these five subsets, i.e., each of the top five models described above was further trained on each of the five subsets. During transfer learning, the weights of each model are trainable without freezing any layer, and the architecture of the neural network and hyperparameters (such as learning rate, dropout rate, and so on) remains the same as before. Therefore, in transfer learning, all the weights of the top five models were further trained on the five subsets separately. Then, there were 25 trained models, and we called them length-dependent models (2dRNA-LD). For prediction, according to the length of a target sequence, we chose the five length-dependent models of the corresponding length range to predict it, and the final result was also averaged over the output of these five models.

In addition, we also performed transfer learning on the PDB-based dataset TR1_neat for the top five 2dRNA-LID models. The trained model was called 2dRNA-LID-PDB, validated on VL1_neat, and tested on TS1_neat. Similarly, for the target sequence, the prediction result was also the average of the output of these five models. Furthermore, since the training set TR1_neat only had 120 RNA sequences, and there were 104 sequences whose length was less than 100 (13 sequences for 100∼200, 1 sequence for 300∼400, and 2 sequences for 400∼500), there were not enough data for training the length-dependent model for all length intervals. Therefore, we retrained the length-dependent model for the length interval of less than 100 nucleotides only, and the trained model was called 2dRNA-LD-PDB.

#### 3.2.4. Performance Measure

The accuracy of the base pairs between the prediction and native structures is calculated to estimate the performance of our models. TP (true positive) is used to denote the number of correctly predicted base pairs, FP (false positive) is the incorrect results, and FN (false negative) represents those base pairs that are in the native structure, but not in the prediction structure. The precision (PPV) and sensitivity (STY) are defined as follows (Equation (1)) [42]:

(1) $$\mathrm{PPV}=\frac{\mathrm{TP}}{\mathrm{TP}+\mathrm{FP}},\;\mathrm{STY}=\frac{\mathrm{TP}}{\mathrm{TP}+\mathrm{FN}}$$

where STY measures the ability to find the positive base pairs and PPV measures the ability of not predicting false positive base pairs.

We also use MCC (Matthews correlation coefficients, Equation (2)) to comprehensively evaluate the prediction results [43]; for this, STY and PPV could not be satisfied simultaneously when comparing the accuracy of the prediction results, and MCC can be treated as the balanced measure between PPV and STY.

(2) $$\begin{array}{ll} \mathrm{MCC} & =\frac{\mathrm{TP}\times \mathrm{TN}-\mathrm{FP}\times \mathrm{FN}}{\sqrt{\left(\mathrm{TP}+\mathrm{FP}\right)\left(\mathrm{FP}+\mathrm{TN}\right)\left(\mathrm{TN}+\mathrm{FN}\right)\left(\mathrm{FN}+\mathrm{TP}\right)}}\\ & \approx \sqrt{\mathrm{STY}\times \mathrm{PPV}} \end{array}$$

## 4. Conclusions

In this paper, we proposed a length-dependent model to improve the performance of RNA secondary structure prediction using deep learning. 2dRNA was used to do this. Furthermore, we used a larger bpRNA dataset and applied a grid search for the hyperparameters of the network. The results show that the length-dependent model performs better than the length-independent model. In fact, it is better to use a type-dependent model, since each type of RNA usually has similar secondary and tertiary structures, and so it is easier for the learning of the deep learning model. However, the number of RNAs of each type is very different and some types only had very few RNAs in the training set. Even for the length-dependent model, RNAs with long sequences are very limited in the training set. As the number of RNAs with known secondary structures increases, the performance of the length-dependent model should also become better.

## Figures and Tables

**Table 1:** The performance of 2dRNA on the validation set VL0 and testing set TS0.

Method (Pairing Cutoff)	VL0			TS0		
	STY	PPV	MCC	STY	PPV	MCC
SPOT-RNA	0.563	0.712	0.632	0.560	0.709	0.629
MXfold2	0.668	0.513	0.576	0.674	0.522	0.583
E2Efold	0.110	0.127	0.112	0.119	0.144	0.126
2dRNA-LID (0.5)	0.552	0.734	0.619	0.545	0.725	0.611
(0.3)	0.613	0.714	0.650	0.610	0.714	0.648
(0.1)	0.674	0.661	0.660	0.670	0.662	0.659
2dRNA-LD (0.5)	0.562	0.769	0.639	0.553	0.752	0.627
(0.3)	0.633	0.768	0.683	0.621	0.759	0.672
(0.1)	0.694	0.708	0.694	0.686	0.702	0.687

**Table 2:** Performances of 2dRNA on TS0 (pairing cutoff = 0.5).

Length Interval	Sequence Number in TR0	2dRNA-LID			2dRNA-LD		
		STY	PPV	MCC	STY	PPV	MCC
≤100	4859	0.632	0.746	0.676	0.639	0.775	0.691
101∼200	4278	0.503	0.690	0.571	0.496	0.711	0.576
201∼300	758	0.443	0.744	0.554	0.463	0.798	0.579
301∼400	626	0.384	0.781	0.527	0.476	0.786	0.591
401∼500	162	0.293	0.729	0.441	0.350	0.791	0.508
Total or Mean	10,814	0.545	0.725	0.611	0.553	0.752	0.627

**Table 3:** Performances of different methods on TS0 (divided by length interval, pairing cutoff = 0.5).

Method	STY					PPV					MCC				
	100	200	300	400	500	100	200	300	400	500	100	200	300	400	500
IPknot	0.623	0.494	0.426	0.414	0.405	0.582	0.449	0.420	0.474	0.467	0.590	0.460	0.416	0.435	0.432
RNAfold	0.627	0.561	0.483	0.457	0.467	0.507	0.413	0.347	0.386	0.394	0.551	0.473	0.405	0.414	0.427
SPOT-RNA	0.778	0.584	0.525	0.414	0.376	0.650	0.517	0.533	0.533	0.505	0.702	0.539	0.518	0.463	0.433
2dRNA-LD	0.639	0.496	0.463	0.476	0.350	0.775	0.711	0.798	0.786	0.791	0.691	0.576	0.579	0.591	0.508

**Table 4:** Prediction of pseudoknot base pairs.

Method (Pairing Cutoff)	TP	FP	FN	STY	PPV	MCC
SPOT-RNA (0.335)	282	6956	924	0.244	0.058	0.115
2dRNA-LID (0.5)	251	4485	955	0.178	0.047	0.084
(0.3)	334	5701	872	0.235	0.055	0.105
(0.1)	460	7771	746	0.324	0.060	0.133
2dRNA-LD (0.5)	461	4835	745	0.303	0.081	0.144
(0.3)	569	5865	637	0.366	0.085	0.166
(0.1)	728	7556	478	0.520	0.102	0.217

**Table 5:** Performance of 2dRNA including non-canonical base pairs.

Method	Pairing Cutoff	STY	PPV	MCC
	0.5	0.554	0.723	0.615
2dRNA-LID	0.3	0.623	0.709	0.652
	0.1	0.688	0.652	0.663
	0.5	0.568	0.750	0.634
2dRNA-LD	0.3	0.640	0.754	0.679
	0.1	0.710	0.692	0.694

**Table 6:** The performance of the top 5 models on VL0 and TS0.

Model ID		VL0			TS0	
	STY	PPV	MCC	STY	PPV	MCC
85	0.522	0.706	0.592	0.516	0.704	0.587
100	0.540	0.650	0.580	0.536	0.646	0.576
242	0.508	0.703	0.580	0.501	0.687	0.570
243	0.547	0.705	0.608	0.532	0.691	0.592
247	0.542	0.675	0.591	0.549	0.683	0.598

**Table 7:** The hyperparameters of the top 5 models.

Model ID	Batch Size	LSTM Layer	Hidden Vector	Learning Rate	Dropout Rate	Base Channel
85	8	2	128	0.004	0.1	32
100	8	3	128	0.002	0.1	16
242	16	2	128	0.002	0.2	16
243	16	2	128	0.002	0.2	32
247	16	2	128	0.004	0.2	32

**Table 8:** Performance of 2dRNA after transfer learning on the testing set TS1_neat.

Method (Pairing Cutoff)		TS1_Neat		TS1_Neat (Length < 101)		
	STY	PPV	MCC	STY	PPV	MCC
SPOT-RNA (0.335)	0.808	0.787	0.790	0.833	0.782	0.800
2dRNA-LID-PDB (0.5)	0.592	0.837	0.682	0.630	0.808	0.696
(0.3)	0.679	0.867	0.753	0.705	0.858	0.763
(0.1)	0.781	0.814	0.791	0.800	0.815	0.802
2dRNA-LD-PDB (0.5)	0.603	0.835	0.689	0.644	0.810	0.705
(0.3)	0.710	0.877	0.777	0.743	0.872	0.794
(0.1)	0.795	0.815	0.799	0.818	0.818	0.812

## Data Availability

The web server 2dRNA including 2dRNA-LD is available at http://biophy.hust.edu.cn/new/2dRNA (accessed on 20 July 2021).

## Supplementary Materials

The following supporting information can be downloaded, Table S1. The summary of TR0; Table S2. The summary of VL0 dataset; Table S3. The summary of TS0 dataset; Table S4. The summary of TR1_neat dataset; Table S5. The summary of VL1_neat dataset; Table S6. The summary of TS1_neat dataset; Table S7. Performance of 2dRNA-LD (0.1) on test set TS0 for different types; Table S8. Performance of 2dRNA-LD on test set TS0 for each length interval; Table S9. Performance of 2dRNA-LD-PDB on test set TS0.

## Sample Availability Statement

Samples of the compounds are not available form the authors.
