# Supplementary material for: Length-Dependent Deep Learning Model for RNA Secondary Structure Prediction
Source: Molecules. 2022 Feb 2;27(3):1030. doi: 10.3390/molecules27031030 (PMC8838716; doi:10.3390/molecules27031030)
Supplement: Supplementary file 1 [file molecules-27-01030-s001.zip › molecules-1523934-supplementary.pdf]

molecules

Supplementary Materials

Length-Dependent Deep Learning Model for RNA Secondary Structure Prediction

Kangkun Mao, Jun Wang and Yi Xiao *

School of Physics and Key Laboratory of Molecular Biophysics of the Ministry of Education, Huazhong University of Science and Technology, Wuhan 430074, China; mkk@hust.edu.cn (K.M.); junwang@hust.edu.cn (J.W.)
* Correspondence: yxiao@hust.edu.cn

Table S1. The summary of TR0 dataset.

Type	0~100	100~200	200~300	300~400	400~500	Length	Sequences
16sRNA	0	0	0	1	1	400~484	2
5sRNA	9	174	0	0	0	41~139	183
CRISPR	41	0	0	0	0	35~47	41
RNaseP	0	1	28	163	55	189~480	247
groupI	0	0	15	10	17	210~487	42
groupII	74	302	116	0	0	66~257	492
microRNA	1070	520	15	2	0	52~364	1607
Riboswitch	85	238	135	0	0	43~248	458
snRNA	632	670	65	11	1	46~416	1379
tRNA	862	0	0	0	0	42~93	862
tmRNA	0	1	8	183	19	102~433	211
Other	2217	2372	376	256	69	33~498	5290
total	4990	4278	758	626	162	33~498	10814

Table S2. The summary of VL0 dataset.

Type	0~100	100~200	200~300	300~400	400~500	Length	Sequences
16sRNA	0	0	0	1	0	308~308	1
5sRNA	3	16	0	0	0	36~143	19
CRISPR	6	0	0	0	0	35~37	6
RNaseP	0	0	2	21	7	203~454	30
groupI	0	0	3	2	2	218~497	7
groupII	9	44	10	0	0	74~244	63
microRNA	118	59	2	0	0	52~274	179
riboswitch	12	34	15	0	0	45~236	61
snRNA	74	74	6	1	0	55~352	155
tRNA	116	0	0	0	0	66~92	116
tmRNA	0	0	0	15	2	347~421	17
other	266	295	50	28	7	33~447	646
Total	604	522	88	68	18	33~497	1300

Table S3. The summary of TS0 dataset.

Type	0~100	100~200	200~300	300~400	400~500	Length	Sequences
16sRNA	0	0	0	1	0	344~344	1
5sRNA	3	26	1	0	0	94~242	30
CRISPR	7	0	0	0	0	28~38	7
RNaseP	0	0	1	18	4	278~432	23
groupI	0	0	2	3	5	234~454	10
groupII	5	38	12	0	0	75~241	55
microRNA	119	79	3	0	0	52~238	201
riboswitch	13	18	17	0	0	43~236	48
snRNA	67	69	8	4	0	56~386	148
tRNA	129	0	0	0	0	57~93	129
tmRNA	0	0	1	21	3	291~437	25
other	243	290	49	29	17	22~499	628
total	586	520	94	76	29	22~499	1305

Table S4. The summary of TR1_neat dataset.

Type	Sequences	Minimumlength	Maximumlength
5sRNA	3	35	122
tRNA	36	34	99
tmRNA	1	68	68
mRNA	1	49	49
riboswitch	20	33	125
RNaseP	2	46	414
group-II	2	388	418
ribosome	3	37	57
other	52	35	186
Total	120	33	418

Table S5. The summary of VL1_neat dataset.

Type	Sequences	Minimumlength	Maximumlength
5sRNA	1	121	121
tRNA	13	35	122
riboswitch	5	34	127
other	11	36	159
Total	30	34	159

Table S6. The summary of TS1_neat dataset.

Type	Sequences	Minimumlength	Maximumlength
5sRNA	3	34	122
tRNA	17	69	92
tmRNA	1	63	63
mRNA	3	38	47
riboswitch	18	41	174
RNaseP	2	34	160
group-I	1	192	192
group-II	1	70	70
ribosome	2	33	55
other	19	34	145
total	67	33	192

Table S7. Performance of 2dRNA-LD (0.1) on test set TS0 for different types.

Type	Sequences	Length	STY	PPV	MCC
16sRNA	1	344~344	0.397	0.592	0.484
5sRNA	30	94~242	0.769	0.757	0.761
CRISPR	7	28~38	0.841	0.806	0.821
RNaseP	23	278~432	0.817	0.835	0.824
groupI	10	234~454	0.492	0.704	0.581
groupII	55	75~241	0.718	0.740	0.726
microRNA	201	52~238	0.744	0.675	0.703
riboswitch	48	43~236	0.607	0.655	0.623
snRNA	148	56~386	0.837	0.806	0.818
tRNA	129	57~93	0.628	0.617	0.611
tmRNA	25	291~437	0.936	0.970	0.952
other	628	22~499	0.717	0.737	0.725
total	1305	22~499	0.686	0.702	0.687

Table 8. Performance of 2dRNA-LD on test set TS0 for each length interval.

Pairing cutoff: 0.5				
**Sequences Interval**	**Sequences**	**STY**	**PPV**	**MCC**
0~100	586	0.639	0.775	0.691
100~200	520	0.496	0.711	0.576
200~300	94	0.463	0.798	0.579
300~400	76	0.476	0.786	0.591
400~500	29	0.350	0.791	0.508
total	1305	0.553	0.752	0.627
**Pairing cutoff: 0.3**				
**Sequences Interval**	**Sequences**	**STY**	**PPV**	**MCC**
0~100	586	0.697	0.796	0.734
100~200	520	0.573	0.713	0.623
200~300	94	0.534	0.754	0.617
300~400	76	0.553	0.800	0.645
400~500	29	0.419	0.738	0.544
total	1305	0.621	0.759	0.672
**Pairing cutoff: 0.1**				
**Sequences Interval**	**Sequences**	**STY**	**PPV**	**MCC**
0~100	586	0.745	0.763	0.748
100~200	520	0.647	0.642	0.637
200~300	94	0.621	0.655	0.631
300~400	76	0.644	0.724	0.675
400~500	29	0.520	0.646	0.576
total	1305	0.686	0.702	0.687

Table S9. Performance of 2dRNA-LD-PDB on test set TS0.

Pairing cutoff: 0.5				
**Sequences Interval**	**Sequences**	**STY**	**PPV**	**MCC**
0~100	586	0.612	0.751	0.664
100~200	520	0.384	0.655	0.479
200~300	94	0.287	0.690	0.422
300~400	76	0.261	0.713	0.411
400~500	29	0.193	0.674	0.332
total	1305	0.468	0.704	0.551
**Pairing cutoff:0.3**				
**Sequences Interval**	**Sequences**	**STY**	**PPV**	**MCC**
0~100	586	0.686	0.749	0.707
200~300	520	0.483	0.636	0.538
200~300	94	0.414	0.633	0.498
300~400	76	0.368	0.642	0.477
400~500	29	0.281	0.534	0.377
total	1305	0.558	0.685	0.604
**Pairing cutoff:0.1**				
**Sequences Interval**	**Sequences**	**STY**	**PPV**	**MCC**
0~100	586	0.744	0.695	0.711
100~200	520	0.569	0.533	0.543
200~300	94	0.528	0.481	0.499
300~400	76	0.486	0.498	0.487
400~500	29	0.396	0.397	0.394
total	1305	0.636	0.597	0.609
